# Promoting Effect of Ce and La on Ni–Mo/δ-Al_2_O_3_ Catalysts in the Hydrodeoxygenation of Vanillin

**DOI:** 10.1021/acs.energyfuels.4c00898

**Published:** 2024-05-18

**Authors:** Tove A. Kristensen, Christian P. Hulteberg, Reine L. Wallenberg, Omar Y. Abdelaziz, Sara Blomberg

**Affiliations:** † Division of Chemical Engineering, Department of Process and Life Science Engineering, Lund University, Lund SE-221 00, Sweden; ‡ Hulteberg Chemistry & Engineering AB, Malmö SE-212 25, Sweden; § Centre for Analysis and Synthesis/nCHREM, Lund University, Lund SE-221 00, Sweden; ∥ Department of Chemical Engineering, King Fahd University of Petroleum & Minerals, Dhahran 31261, Saudi Arabia; ⊥ Interdisciplinary Research Center for Refining & Advanced Chemicals, King Fahd University of Petroleum & Minerals, Dhahran 31261, Saudi Arabia

## Abstract

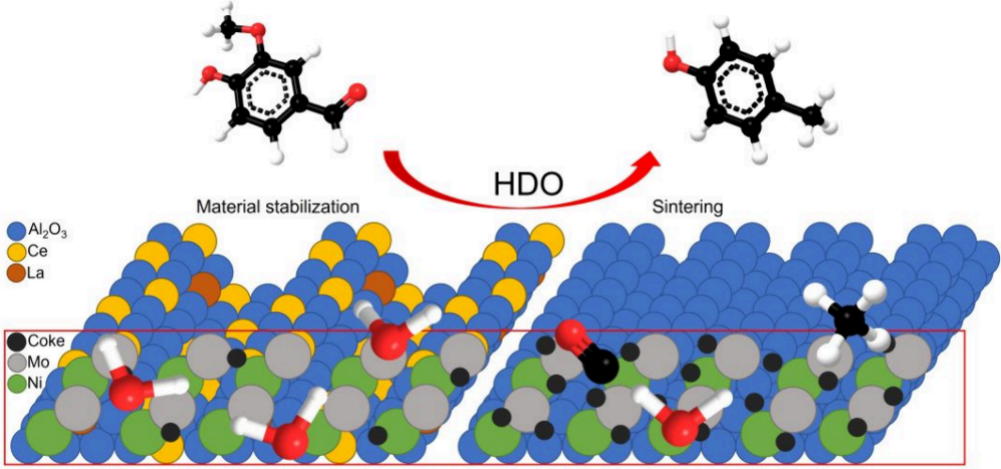

A crucial aspect of adding an economical and environmental dimension to the upgrading of bio-oils is to develop catalysts with enhanced and prolonged activity. In the present study, the effect of doping δ-alumina (Al_2_O_3_) with oxides of cerium (Ce) and lanthanum (La) before thermal treatment was investigated. The performance of such an Al_2_O_3_-supported nickel–molybdenum (Ni–Mo) catalyst was evaluated by studying the selectivity for the direct hydrodeoxygenation (HDO) of vanillin to cresol under continuous-flow conditions. In addition, the effect of adding H_2_S during catalyst activation and/or performance tests was also evaluated. Overall, enhanced performance of the doped NiMo catalyst in the HDO process has been demonstrated and an increased selectivity for cresol via direct HDO observed. The advantage of adding La and Ce is supported by the characterization results, where less sintering and enhanced pore diameter of the doped Al_2_O_3_ were observed after thermally inducing the transformation from the δ to θ phases. The improved characteristics and prolonged activity of the doped Al_2_O_3_ were also deduced by the lower acidity of the catalyst, which resulted in reduced coke formation during the HDO process.

## 1 Introduction

Lignin is an underutilized lignocellulosic feedstock that has the potential to produce renewable liquid fuels. Lignin is a multifunctional oxygenated aromatic biopolymer with a complex three-dimensional structure.^1^ The lignin energy-rich aromatic monomer constituents are highly suitable as a resource for producing bio-oil fuels, and enabling their utilization is of great environmental interest.^2^ However, isolated, purified, and depolymerized liquefied lignin oils contain high amounts of oxygen and are not appropriate for direct use (e.g., due to low heat values, thermal instability, and storage limitations) but require further quality upgrading treatment before they can be used as a fossil-substituted alternative.^3,4^

An efficient upgrading method for lignin oils is catalytic hydrodeoxygenation (HDO).^5,6^ HDO is a hydrotreatment process adapted from other well-known and industrially applied heteroatomic removal processes. Sulfided Mo promoted with Ni (or Co) on γ-Al_2_O_3_ support is the most frequently used and reported catalyst active for the HDO of bio-oils.^7−9^ In addition to its efficiency in the HDO processing of bio-oils, the Ni(or Co)Mo/γ-Al_2_O_3_ catalyst is advantageous as it is already used in hydrotreatment processes at oil refineries.^10^ However, a challenging aspect of using the sulfided NiMo/Al_2_O_3_ catalyst for the HDO process of bio-oils is to preserve the stability of its recognized active sites (MoS_2_-slab edges). This, as the necessary accessibility of sulfur to preserve the sulfided state in bio-oils, can be limited and inadequate in that respect. For that reason, development of nonsulfided catalysts for the HDO of bio-oils is important and promoted MoO_3_ has been suggested to be a promising catalyst.^11,12^

Overall, the catalyst is a decisive aspect of the product distribution resulting from the HDO processing of bio-oils. Many factors such as surface textural and structural properties, along with metal dispersion and agglomeration, and chemical properties of active sites (e.g., acidity-basicity and redox properties) affect the catalyst suitability.^6^ This, in conjunction with the support, which is also a crucial aspect, contributes to the overall catalytic performance. The support should enable sufficient diffusion of reactants and products and withstand structural alteration caused by used process conditions.^5,13^

The most critical challenge for enabling the large-scale HDO treatment of bio-oils is to maintain the performance of the catalyst. Balanced concentration and strength of active acid sites and steric hindrance are all conjunctive affecting parameters. It is a major challenge to develop a catalyst with properties not leading to short-term deactivation in the HDO process due to pore blockage by, e.g., carbon deposit, and negatively affecting structural composition due to the need for severe processes.^6^

The utilization of rare earth metals, such as La and Ce, for industrial hydrocarbon processing catalysts has been shown to decrease coke formation, thermally stabilize structural composition, and hence improve catalytic activity. The advantage of doping supports with rare earth metals is attributed to the possibility to affect the electrostatic structure of such, e.g., acidity and metal interactions.^14^ The effects of rare earth metals in catalyst–support systems used in, e.g., reforming and partial oxidation processes, have been recognized.^15,16^ Less attention has been given to the corresponding possible enhancement of the HDO process and the upgrading of lignin oils. Yet, beneficial tendencies have been reported, as for some challenging aspects. Sangnikul et al.^8^ investigated the effect of adding Ce (2–8 wt %) on NiMo/γ-Al_2_O_3_ for the HDO of guaiacol and bio-oil and did observe overall beneficial effects (enhanced metal distribution and catalytic performance). However, in contrast to when performing the HDO over guaiacol, Sangnikul et al.^8^ did not observe a decrease in coke deposition on the catalyst surface when performing HDO of a bio-oil feedstock and metal leaching was proposed as the reason for this. Metal leaching is a possible challenging aspect for using rare earth metals with basic characteristics as promoters for the HDO process of bio-oils with acidic properties. Escobar et al.^17^ investigated Pt/Al_2_O_3_ catalysts modified with varying loadings of La (0.5–8 wt %), for the HDO of guaiacol. A La concentration of 1 wt % showed significantly higher activity compared to pristine Al_2_O_3_. The beneficial effect of La was, however, diminished at high La loadings. Escobar et al.^17^ did, in addition, also conclude that the addition of La affected the HDO reaction route, which was attributed to the La contribution of basic surface sites. Furthermore, the impact of the impregnation order of Ce and La on NiCu/Al_2_O_3_ catalysts was investigated by Vandevyvere et al.^18^ In general, the addition of Ce resulted in beneficial performance due to enhanced metal interactions whereas the addition of La showed beneficial stabilization attributes only when added sequentially before Ni and Cu. The addition of La as a sequential last step resulted in lowering the activity as it blocked the active sites.^18^ Overall, a more comprehensive understanding and further investigation of the effects of using rare earth metals, such as Ce and La, in the HDO of bio-oils are encouraged and of interest.

Considering the above aspects, the aim of this study is to investigate the possibility of enabling prolonged and enhanced catalytic activity of the NiMo/Al_2_O_3_ catalyst by the use of La and Ce as dopants. The insights can be used to improve the industrial hydrotreatment technology for upgrading lignin oils, a critical step toward an environmentally friendly alternative to fossil-derived fuels. Herein was the interest to apply the present knowledge from, e.g., steam reforming and partial oxidation process, regarding adding rare earth metals to Al_2_O_3_-supported catalysts, but for the HDO of a lignin model compound.

## 2 Experimental Section

### 2.1 Catalyst Synthesis

Both the Ce- and La-doped support materials and the different NiMo catalysts were prepared using incipient wetness impregnation. δ-Al_2_O_3_ extrudates (Sasol Germany GmbH, Germany) were used in the examination of the textural support characteristics. The extrudates were crushed and sieved into particles with a size range between 1 and 2 mm before catalyst production. δ-Al_2_O_3_ were impregnated simultaneously with 1 wt % of both Ce (cerium(III)nitrate hexahydrate 99.5%, Treibacher Industrie AG) and La (lanthanum(III) nitrate hexahydrate, 99.9% (REO), Alfa Aesar). The Ce- and La-impregnated δ-Al_2_O_3_ was dried at 120 °C for 4 h and subsequentially thermally treated at 1100 °C for 4 h (both steps with a temperature ramp of 2 °C/min). Equivalent amounts of 8.0 wt % Mo (ammonium molybdate tetrahydrate, ACS Reagent, Sigma-Aldrich) and 3.5 wt % Ni (nickel(II) nitrate hexahydrate, Sigma-Aldrich) were impregnated in separate subsequent steps on both the reference δ-Al_2_O_3_ support and the pretreated Ce- and La-doped support. Citric acid monohydrate was added to the solution when Ni was impregnated (0.7 molar ratio to Ni). Each impregnation step included drying at 120 °C for 4 h and calcination at 500 °C for 4 h. All elevated temperatures were reached with a set temperature ramp of 2 °C/min.

### 2.2 Catalyst Characterization

The effect on the textural properties by doping δ-Al_2_O_3_ with Ce and La was evaluated on fresh samples using N_2_ physisorption and X-ray diffraction (XRD), whereas structural characteristics were analyzed using chemisorption and temperature-programmed desorption (TPD), both with ammonia (NH_3_) as the probing adsorbent. Metal distribution on fresh catalysts was evaluated using scanning electron microscopy (SEM). Carbon and sulfur deposition on the used catalysts was evaluated using elemental analysis.

#### 2.2.1 N_2_ Physisorption

The textural properties of the supports and catalysts were evaluated (approximately 0.5 g) by determination of the N_2_ adsorption–desorption isotherms using a 3Flex instrument (Micromeritics, Norcross, Georgia, U.S.). The specific surface area was determined using the Brunauer–Emmett–Teller (BET) equation, and the pore characteristics (size and volume) were determined using the Barrett–Joyner–Halenda (BJH) method.^19,20^ The samples were degassed under vacuum conditions at 90 °C for 30 min and subsequently at 250 °C for 220 min (ramp 10 °C/min) prior to N_2_ physisorption analysis.

#### 2.2.2 X-ray Diffraction

Powder X-ray diffraction measurements were done using monochromatic Cu K_α_ radiation (λ ≈ 0.15418 nm) in a STOE STADI MP X-ray diffractometer coupled with a Mythen 1K detector (STOE & Cie GmbH, Darmstadt, Germany). A collection time of 5 s and a step size of 0.12° were used. The diffractograms were analyzed using rietveld refinement and the BGMN-based Profex software.^21^

#### 2.2.3 NH_3_ Chemisorption and Temperature-Programmed Desorption

The total acidity of the supports and catalysts was determined with chemisorption, whereas the strength of their nature was evaluated using TPD. Both evaluation techniques were done with NH_3_ and performed on a 3Flex instrument (Micromeritics, Norcross, Georgia). Samples of ∼0.27 g were analyzed. Static chemisorption was performed at 100 °C, whereas TPD was performed from 50 to 700 °C (ramp 10 °C/min). The catalysts were reduced prior to the chemisorption analysis at 550 °C (ramp 10 °C/min) for 30 min. A gas flow rate (10% H_2_ in Ar) of 10 mL/min was used. These catalyst samples were afterward used for TPD analysis. Supports were not reduced prior to analysis. In the TPD analysis, the samples were dosed with NH_3_ for 15 min at 50 °C, after which helium was used as gas (10 mL/min) during heating.

#### 2.2.4 H_2_ Temperature-Programmed Reduction

The reducibility of the catalysts was investigated by dynamic chemisorption of H_2_ using a 3Flex instrument (Micromeritics, Norcross, Georgia, U.S). Samples of ∼0.27 g were analyzed using a gas flow (10% H_2_ in Ar) of 10 mL/min over a temperature range from ambient to 1000 °C (ramp 10 °C/min).

#### 2.2.5 Elemental Analysis

Carbon and sulfur depositions on the used catalysts (sample weights of approximately 1–2 mg) were measured with a vario MICRO cube instrument (Elementar Analysensysteme GmbH, Langenselbold, Germany) with a thermal conductivity detector (TCD). Gas used during the measurements was oxygen (10 mL/min) and helium (20 mL/min). The column temperature was 1100 °C.

#### 2.2.6 Scanning Electron Microscopy

A field emission scanning electron microscope, JEOL JSM-6700F (JEOL, Akishima, Tokyo, Japan) instrument with a field emission gun, was used to obtain high-resolution images of the fresh catalysts. An attached energy-dispersive X-ray spectrometer system (80 mm^2^ X-Max, Oxford Instruments, Abingdon, UK) was used for elemental mapping analysis of the catalysts. A carbon layer of approximately 40 nm was deposited on the samples prior to analysis to enhance the surface conductivity.

#### 2.2.7 X-ray Fluorescence

The relative metal ratios were determined using a PANalytical Epsilon 3XL X-ray spectrometer (Malvern PANalytical Ltd., Malvern, UK). Approximately 1 g of each catalyst was analyzed four times.

### 2.3 HDO Activity Tests

The HDO activity tests of vanillin were performed in a continuous-flow fixed-bed reactor in accordance with the procedure described in detail in our previously reported study by Kristensen et al.^22^ In brief, the tests were performed in the gas phase at 314 °C, 5 barg, and with a weight-hourly space velocity (WHSV) of 35 h^–1^. These conditions were established to be the optimal conditions for maximal selectivity to cresol over the reference NiMo catalyst in the equivalent HDO system.^22^ The catalysts were activated in the reactor prior to each performance test at 400 °C (temperature ramp 2 °C/min) and at a total pressure of 8 bar using either pure H_2_ or 1% H_2_S in H_2_, both with WHSV of 400 h^–1^. Activation with pure H_2_ is herein referred to as reduction, while sulfidation is referred to when the gas with 1% H_2_S in H_2_ was used. Either gas was also used during the catalytic performance test. Hence, a total of eight tests were performed. The liquid produced for 30 min prior to the set time of 8 h was collected and analyzed using a gas chromatograph, 456-GC (SCION Instruments, Goes, The Netherlands), with a Rtx DHA-50 column, 50 m × 0.2 mm (Restek, Center County, Pennsylvania), coupled to a flame ionization detector (FID). Gas bags were used to collect the produced gas under 20 min prior to the set experimental time, which was then analyzed in an equivalent 456-GC, coupled to a TCD.

## 3 Results and Discussion

### 3.1 Effect of the Addition of Ce and La on Support and Catalyst Characteristics

**Figure 1:**
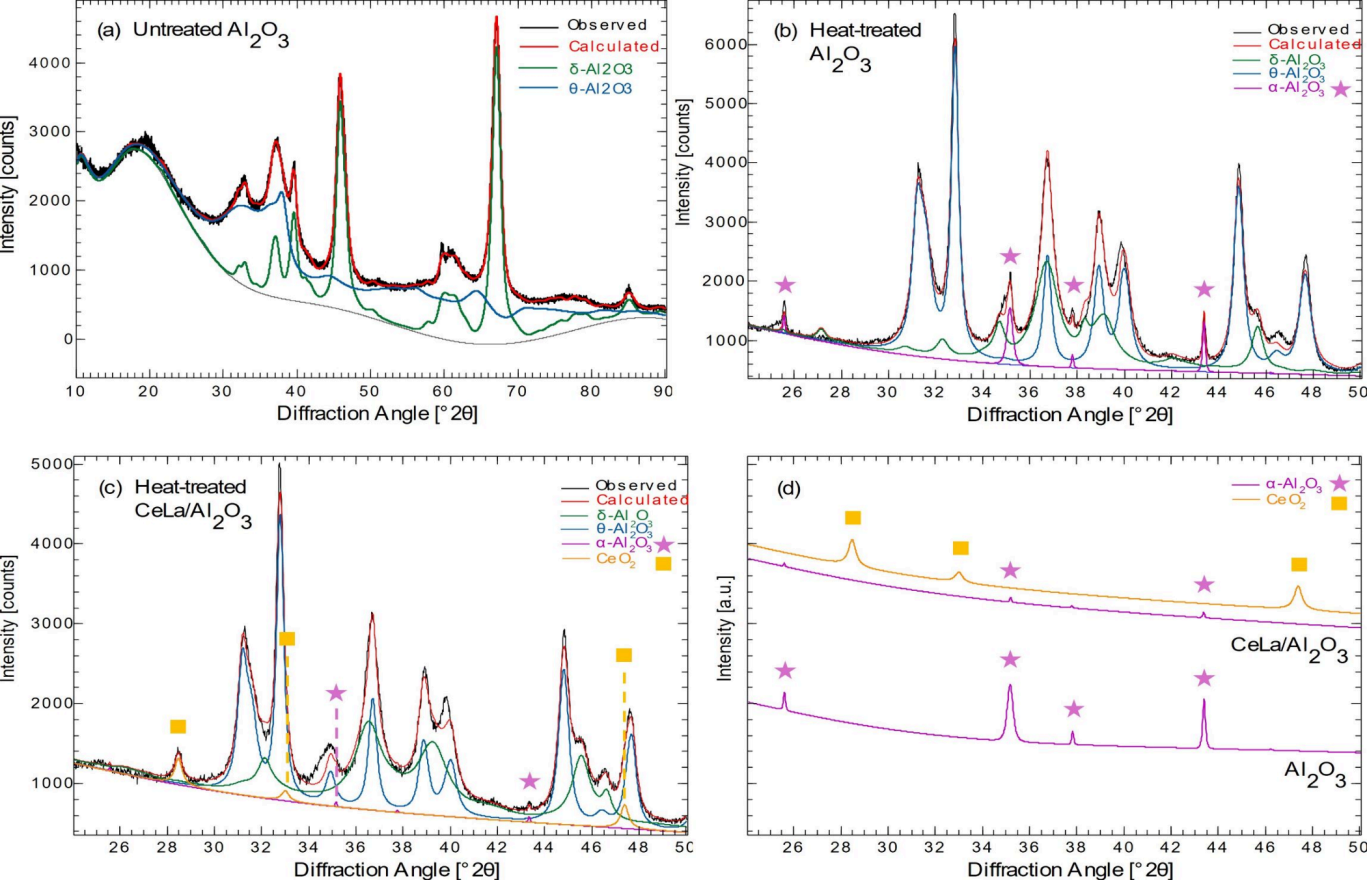
XRD patterns for (a) untreated Al_2_O_3_ support, (b) heat-treated Al_2_O_3_, (c) heat-treated Ce- and La-doped Al_2_O_3_, and (d) specific α-structure peaks and CeO_2_ from diffractograms in (b) and (c).

The results from N_2_ physisorption and XRD analysis of the supports show that Al_2_O_3_ doped with Ce and La sinters less than the reference Al_2_O_3_ and forms pores with increased diameter after being thermally exposed to 1100 °C and transformed to the designated θ-phase. Table 1 shows the surface area, pore volume, and pore diameter of the reference Al_2_O_3_ support and the heat-treated Ce- and La-doped and undoped Al_2_O_3_, and Figure 1 shows the resulting crystal structure. The pore diameter increases in conjunction with the crystal phase change from the initial δ- to θ-phase for both heat-treated samples when increasing the temperature to 1100 °C. However, the XRD confirms a partial transition to the α-phase in the undoped Al_2_O_3_, which is not observed for the Ce- and La-doped Al_2_O_3_ (Figure 1). This, in combination with the fact that the Ce- and La-doped Al_2_O_3_ shows the largest increase in the pore diameter yet having approximately the same surface area as the undoped Al_2_O_3_, suggests an overall material stabilization effect by the Ce and La addition. The resulting data for the δ-Al_2_O_3_ sample are consistent with the recognized temperature-induced phase transformation and associated sintering of Al_2_O_3_ found in the literature. It has previously been reported that δ-Al_2_O_3_ transforms to the designated metastable θ-phase at approximately 950 °C and further to the stable α-phase in the range of 1050–1200 °C, and a profound sintering and surface area loss in that temperature range.^23,24^

**Table 1:** Textural Properties and the Crystal Phase of the Reference Pristine Al_2_O_3_ Support and the Thermally Treated (1100 °C) Ce- and La-Doped and Undoped Al_2_O_3_ Samples

sample	temperature (°C)	BET surface area (m^2^/g)	BJH desorption pore volume (mL/g)	BET pore diameter (nm)	designated crystal phase
pristine Al_2_O_3_	ambient	115	0.81	22	δ
Al_2_O_3_	1100	66	0.52	24	θ
CeLa/Al_2_O_3_	1100	65	0.58	28	θ

Furthermore, XRD analysis revealed the formation of CeO_2_ (Figure 1c). However, no indication of the formation of La_2_O_3_ is observed and La is therefore suggested to strongly interact with Al_2_O_3_. LaAlO_3_ is reported to be formed at 800 °C,^25^ whereas the formation of CeAlO_3_ needs a higher temperature.^26^ Church et al.^27^ detected that Ce impregnated on γ-Al_2_O_3_, contrary to La, segregated as CeO_2_ after 24 h at 1100 °C. La is therefore suggested to be the metal being dominant for the material stabilization. This is in agreement with other studies investigating the effect of adding rare earth metals to Al_2_O_3_ where rare earth metals have been shown to decrease the number of nucleation sites of Al_2_O_3_ and, as a result, hinder sintering and retard phase transformation.^15,25,27,28^

**Figure 2:**
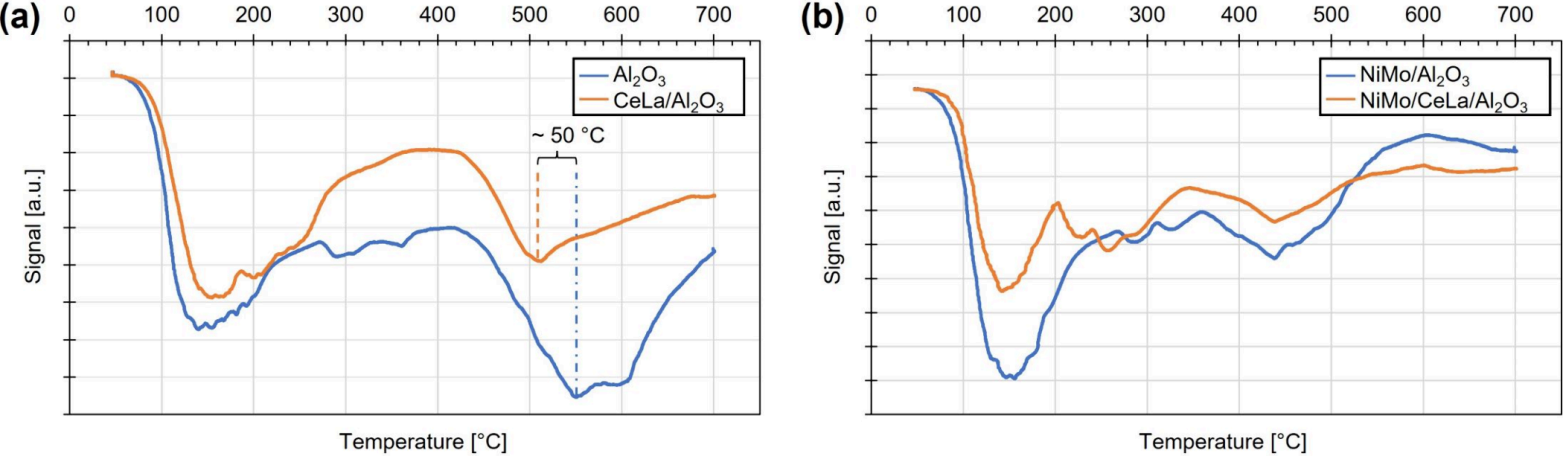
NH_3_-TPD profiles of (a) δ-Al_2_O_3_ and Al_2_O_3_ doped with Ce and La and (b) NiMo/δ-Al_2_O_3_ and NiMo/CeLa/Al_2_O_3_ catalysts.

In correlation with the textural properties, the number and strength of the acidic sites on both the support and the catalyst are important factors that affect the overall performance of the catalyst. The acidity is recognized to be a prerequisite for carbon polymerization, leading to deactivation of the catalyst. Carbon formation on the surface can therefore be suppressed by reducing the acidity of such. The acidity was analyzed by chemisorption and TPD, with NH_3_ as a probing molecule. The results show that the reference δ-Al_2_O_3_ support and catalyst have a total acidity of 1.11 and 4.55 cm^3^/g, respectively, whereas the corresponding values are 0.62 and 2.82 cm^3^/g for the Ce- and La-doped versions. Figure 2 shows the results from the performed TPD for (a) the supports and (b) the catalysts. In general, two NH_3_ desorption peaks, assigned to weaker and stronger acidic sites, respectively, are observed in the TPD profile for the reference δ-Al_2_O_3_ support (Al_2_O_3_ in Figure 2a) and catalyst (NiMo/Al_2_O_3_ in Figure 2b). The effect of adding La and Ce can be detected in the TPD profile as a 50 °C lower desorption temperature of the NH_3_ assigned to the stronger acid sites can be observed. The intensity ratios of the two NH_3_ desorption signals are also affected, interpreted as the Ce- and La-doped samples having fewer strong acid sites. In addition, a third desorption peak at approximately 200–350 °C is detected for the doped NiMo catalyst (NiMo/CeLa/Al_2_O_3_ in Figure 2b), which is suggested to be NH_3_ desorption of Ce located on the support that blocks the stronger acidic sites. The catalysts are reduced prior to the TPD study, which allows for the adsorption of NH_3_ on the Ce as well and hence results in a third desorption peak.

**Figure 3:**
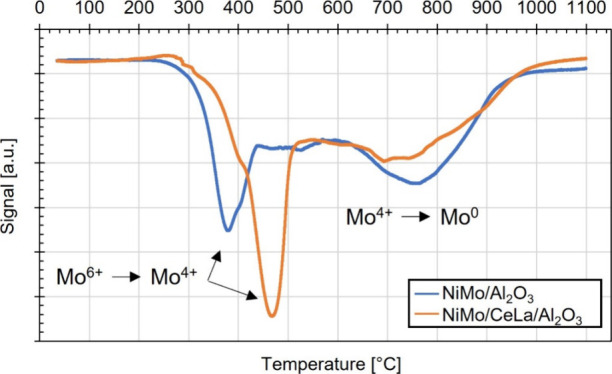
H_2_**-**TPR profiles of the NiMo/δ-Al_2_O_3_ and NiMo/CeLa/Al_2_O_3_ catalysts.

Additional characteristics of the catalysts were examined by using H_2_-TPR. Figure 3 shows the resulting H_2_-TPR profiles for both the reference and doped NiMo catalysts. A distinct partial reduction of MoO_3_ specimen (Mo^6+^ → Mo^4+^) is observed at 380 and 470 °C for the reference and doped NiMo catalysts, respectively. A subsequent reduction of Mo species (Mo^4+^ → Mo^0^) over a broader range at higher temperatures (∼700 °C) can also be distinguished. The observed partial reduction stages are consistent with results presented by Blomberg et al.^29^ in regard to the promotional effect of Ni in NiMo/δ-Al_2_O_3_ catalysts. In addition, an indication of a peak at 400 °C and a tendency of a peak shoulder at 800 to 900 °C for the doped NiMo catalyst can be distinguished (Figure 3). This is suggested to originate from the incorporated Ce oxides. The peak at 400 °C is suggested to be ascribed to surface Ce and the shoulder at 800 to 900 °C to the bulk.^30,31^

The overall reducibility behavior of a catalyst is complex, and the interpretation of the resulting H_2_-TPR profile for the doped NiMo catalyst is challenging, where several characteristics are suggested to be affected by the addition of La and Ce that is not discussed in the section above. First, several studies suggest that Ce changes the electronic properties and enhance the dispersion of the metals, which would result in increased reduction temperatures. This could be a factor of the resulting higher reduction temperature of Mo^6+^ to Mo^4+^ for the doped NiMo catalyst (Figure 3). However, weaker interactions between Ni and Mo would also result in higher reduction temperatures. Blomberg et al.^29^ showed that both Mo/δ-Al_2_O_3_ and Ni/δ-Al_2_O_3_, respectively, reduce at higher temperatures compared to the defined NiMo/δ-Al_2_O_3_. Lastly, the reduction of Ni/δ-Al_2_O_3_ occurs over one broad temperature range (approximately 600 °C) whereas the reduction of Mo/δ-Al_2_O_3_ occurs over two distinct ranges (approximately at 540 and 960 °C).^29^ Hence, it is suggested that the subsequent partial reduction of Mo (Mo^4+^ → Mo^0^), detected as the second reduction peak at approximately 750 °C, is related to interactions between the Mo specimen and Al_2_O_3_. The observed lower intensity of this peak, in turn, indicates fewer interactions between Mo and Al_2_O_3_ for the doped NiMo catalyst.

**Figure 4:**
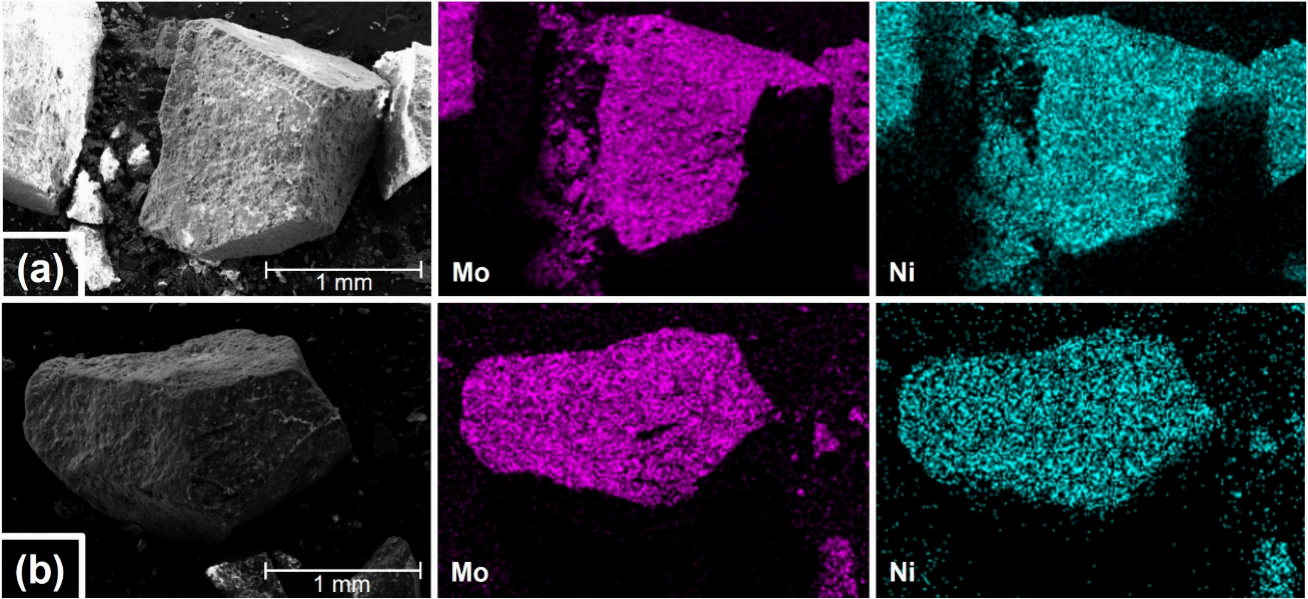
SEM images and EDX elemental distribution of the NiMo catalysts (a) NiMo/δ-Al_2_O_3_ and (b) NiMo/CeLa/Al_2_O_3_.

The metal distribution was investigated by SEM-EDX mapping. Figure 4 shows the SEM and EDX mapping images, and on the millimeter scale, the distributions of Mo and Ni are homogeneous on both the reference catalyst (Figure 4a) and the doped NiMo catalyst (Figure 4b). Furthermore, the XRF analysis revealed that the relative average ratio of Ni and Mo was 0.09 (SD < 0.001) for both the NiMo catalysts. For the doped support, it was revealed that the ratios of La and Ce in relation to the Al_2_O_3_ were 0.05 (SD = 0.02) and 0.06 (SD = 0.02) respectively. We therefore conclude that the addition of Ce and La does not have an impact on overall impregnation.

### 3.2 Hydrodeoxygenation Activity Tests

The catalytic performance of the synthesized NiMo catalysts and the effect of doping δ-Al_2_O_3_ with Ce and La were evaluated with respect to their activity in relation to the sulfur dependency in HDO of vanillin and their selectivity of direct HDO to cresol. In addition, as the major reason for catalyst deactivation in HDO is coke formation, the used catalysts were also evaluated with respect to the surface deposition of carbon and sulfur.

#### 3.2.1 Reaction Network and Product Distribution

**Figure 5:**
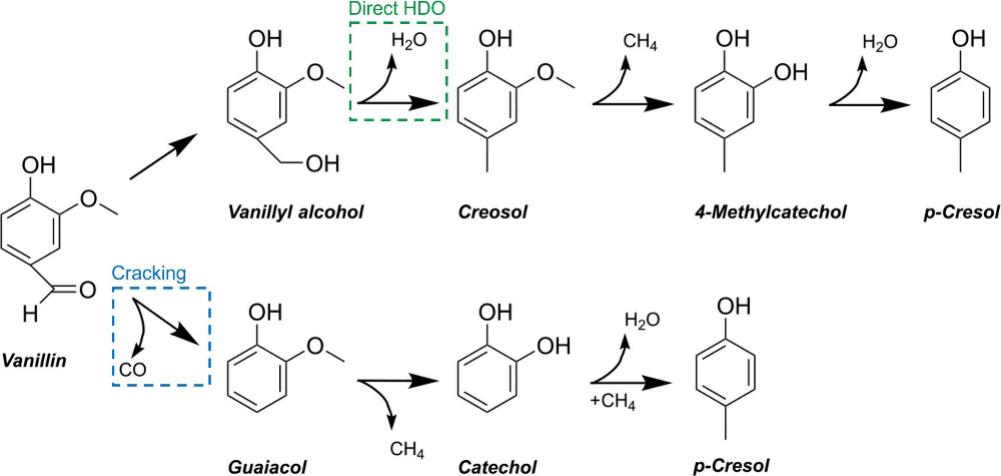
Proposed reaction pathways for the HDO of vanillin.

The selectivity toward cresol in HDO of vanillin is dependent on the synergistic effects of catalyst characteristics and the process conditions. Herein, the reaction pathways, selectivity, and product distribution are evaluated with respect to catalyst characteristics. Figure 5 shows two proposed reaction pathways of the HDO of vanillin. In the direct HDO pathway, the formation of cresol is initiated by hydrogenation and subsequent hydrogenolysis of the aldehyde group, forming creosol via vanillyl alcohol.^32,33^ This is followed by demethylation, hydrogenation, and hydrogenolysis, forming *p*-cresol, via 4-methylcatechol.^34,35^ Demethylation is favored on Al_2_O_3_ compared to demethoxylation.^34^ In the other reaction pathway, the cracking of the C–C bond is the initial step,^7^ forming guaiacol instead of creosol. Cleavage of the C–C bond is promoted by increased temperature and acidity of the catalyst.^7^ Reported reaction pathways from guaiacol are also related to hydrogenation, demethylation, hydrogenolysis, and methylation, and the formation of both *p*- and *o*-cresol is reported as possible reaction products via catechol.^34,36^ The formation of *m*-cresol has been reported not to be favored.^34^

**Figure 6:**
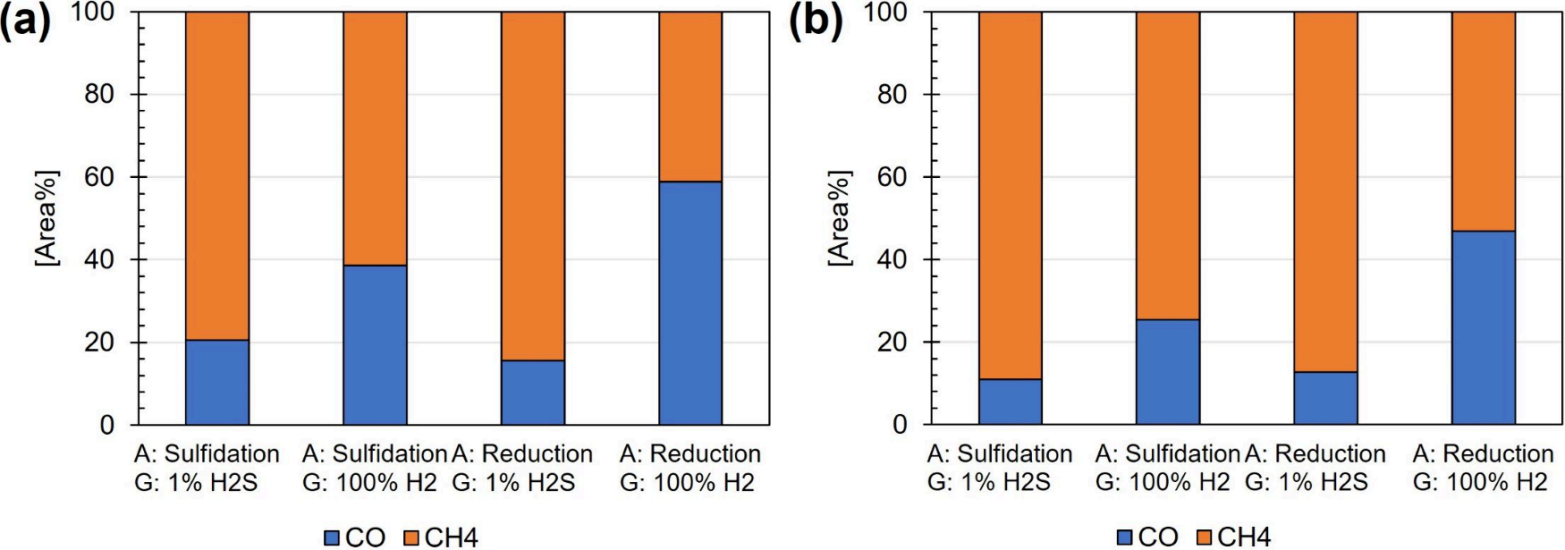
Relative ratios of produced CO and CH_4_ in the gas phase collected, after 8 h (during a 20 min period), in each specified activity test over (a) NiMo/δ-Al_2_O_3_ and (b) NiMo/CeLa/Al_2_O_3_. Varying experimental factors used for each test are presented as A (activation method; sulfidation or reduction) and G (gas feed used during HDO; 100% H_2_ or 1% H_2_S (in 99% H_2_)). Experimental conditions: *T* = 314 °C and *P* = 5 bar_(g)_.

**Figure 7:**
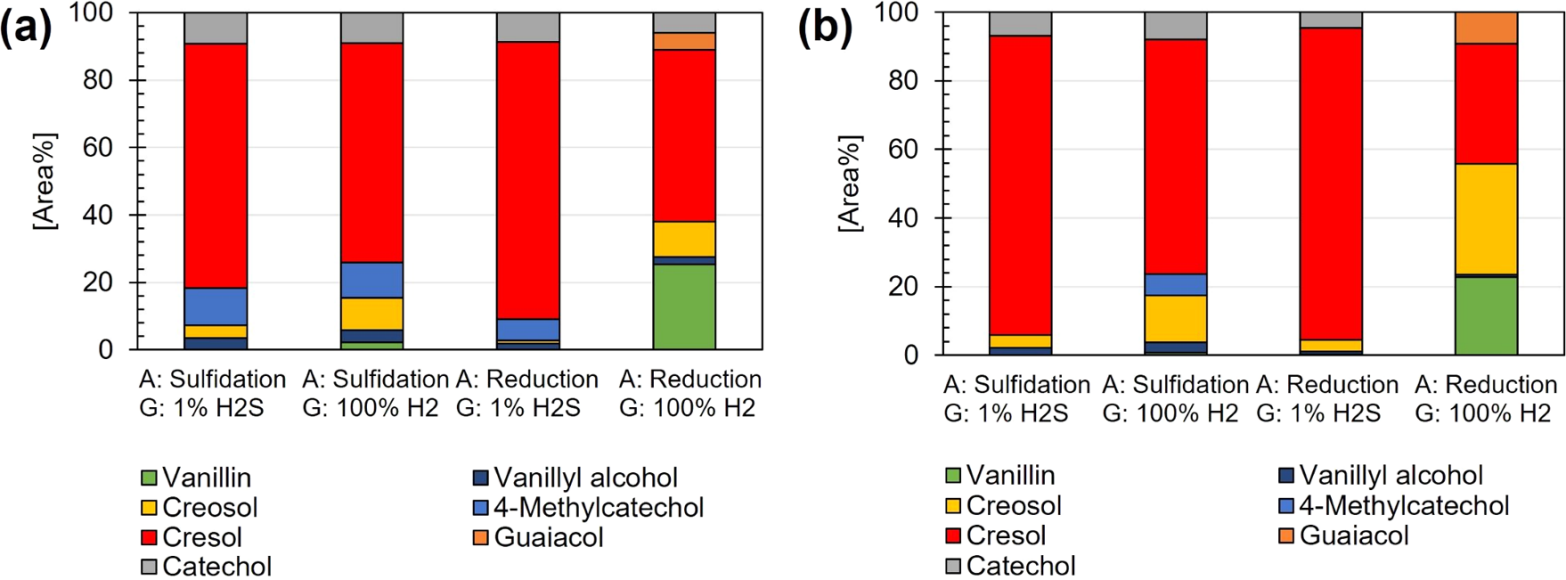
Relative ratios of defined products in liquid samples collected, after 8 h (during a 30 min period), in each specified activity test over (a) NiMo/δ-Al_2_O_3_ (b) NiMo/CeLa/Al_2_O_3_. Varying experimental factors used for each test are presented as A (activation method; sulfidation or reduction) and G (gas feed used during HDO; 100% H_2_ or 1% H_2_S (in 99% H_2_)). Experimental conditions: *T* = 314 °C and *P* = 5 bar_(g)_.

The absolute distribution of the inlet carbon in the outgoing streams for specified activity tests is presented in Table 2. Figure 6 shows the ratio of CH_4_ and CO produced during the specified activity tests for each NiMo catalyst. No other carbon gas component, except for CO and CH_4_, was identified in the gas stream. Figure 7 shows the product distribution in the collected liquid samples during the specified activity tests for each NiMo catalyst with respect to possible products presented in the proposed reaction pathway network (Figure 5). The conversion over time is presented as Supporting Information (Figure S1).

**Table 2:** Absolute Carbon Distribution in the Outgoing Liquid and Gas Streams for the Activity Tests^a^

			carbon distribution [%]	
catalyst	activation method	gas in feed	liquid_out_	gas_out_
NiMo/δ-Al_2_O_3_	sulfidation	1% H_2_S, 99% H_2_	99.7	0.3
NiMo/δ-Al_2_O_3_	sulfidation	100% H_2_	99.8	0.2
NiMo/δ-Al_2_O_3_	reduction	1% H_2_S, 99% H_2_	99.6	0.4
NiMo/δ-Al_2_O_3_	reduction	100% H_2_	99.8	0.2
NiMo/CeLa/Al_2_O_3_	sulfidation	1% H_2_S, 99% H_2_	99.7	0.3
NiMo/CeLa/Al_2_O_3_	sulfidation	100% H_2_	99.7	0.2
NiMo/CeLa/Al_2_O_3_	reduction	1% H_2_S, 99% H_2_	99.6	0.4
NiMo/CeLa/Al_2_O_3_	reduction	100% H_2_	99.8	0.2

a Varying experimental factors used for each test are presented as activation method and gas feed used during HDO. Experimental conditions: *T* = 314 °C and *P* = 5 bar_(g)_.

It was found that higher concentrations of CO are produced when experiments are performed with the reference NiMo catalyst compared with the doped NiMo catalyst (Figure 6). The reference NiMo catalyst contains more acidic sites, as shown in the chemisorption analysis (Figure 2b), compared to the doped NiMo catalyst, and is more prone to crack C–C,^7^ hence resulting in the observed higher CO formation. In accordance with these results and the proposed reaction pathway (Figure 5), enhanced selectivity for direct HDO via hydrogenolysis is suggested for the doped NiMo catalyst compared to the reference catalyst. Interestingly, it seems that neither catalyst is dependent on the activation method for being active in the HDO process but rather on whether sulfur is added to the gaseous feed during the process. The concentration of H_2_S in the collected gas samples was 0.76 and 0.88 mol % for experiments performed over the reference NiMo/δ-Al_2_O_3_ catalyst, using the gas feed containing 1% H_2_S (in 99% H_2_), and when the catalyst was preactivated by defined sulfidation and reduction methods, respectively. Corresponding concentrations of H_2_S in gas samples for the doped NiMo catalyst showed 0.81 and 0.73 mol %, respectively. As expected, no H_2_S was detected in the gas samples for either catalyst when 100% H_2_ was used as the gas feed. The observed sulfur dependency is related to the recognized activity, enhancing the formation of MoS_2_ slabs and associated sulfur vacancies with resulting oxygen adsorbing Mo sites and proton donation possibilities.^11^ We speculate that the observed fewer Mo–O–Al linkages for the doped NiMo catalyst (Figure 3) argue for decreased MoS_2_ lateral sizes and hence more formed MoS_2_ slabs and higher activity. This is in agreement with the detected concentrations of H_2_S in the gas outlet, which indicates a higher sulfidation degree for the doped NiMo catalyst. However, as the conversion of vanillin is close to 100% for both the doped and reference NiMo catalysts when sulfur is added to the feed, the resulting effect of increased sulfidation degree correlated with the number of active MoS_2_ slabs cannot be accurately determined within this scope. A distinct deactivation of both catalysts is observed when no sulfur is added (Figure 7), and the conversion is reduced to 88% after 8 h.

#### 3.2.2 Carbon and Sulfur Deposition on Catalysts

**Figure 8:**
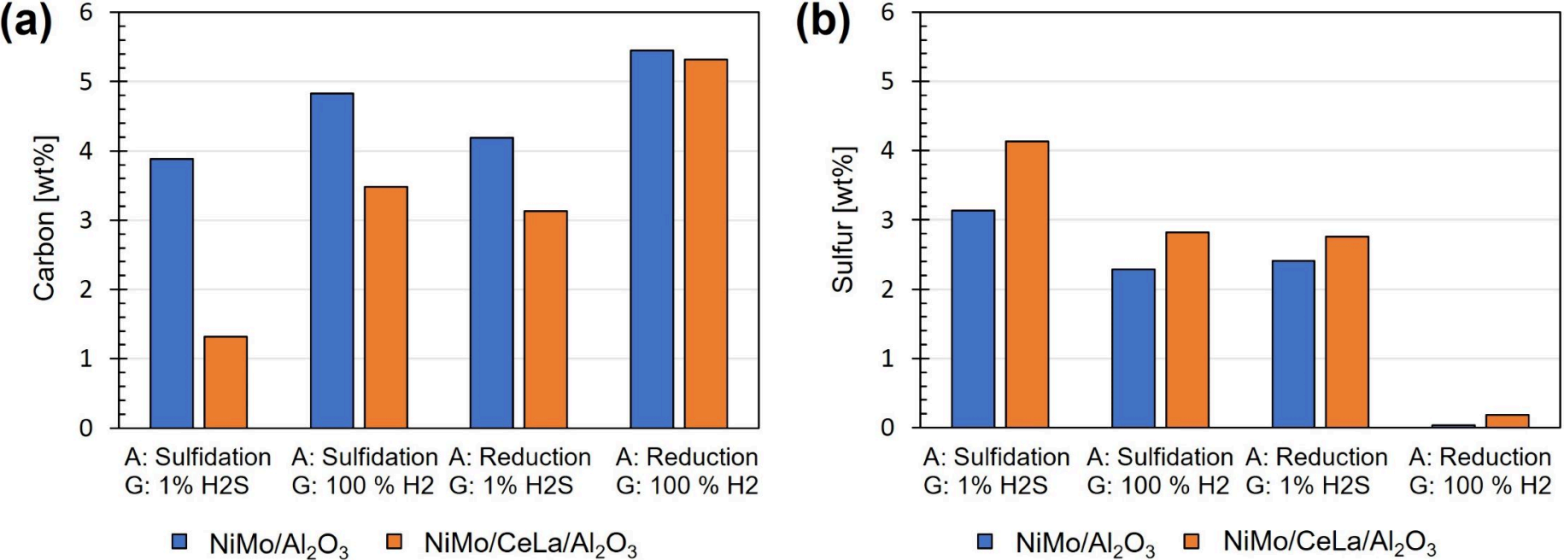
Elemental analysis of the used catalyst and resulting deposition of (a) carbon and (b) sulfur. Varying experimental factors used for each test are presented as A (activation method; sulfidation or reduction) and G (gas feed used during HDO; 100% H_2_ or 1% H_2_S (in 99% H_2_)).

Elemental analysis of catalysts shows less carbon deposition on the doped NiMo catalyst and a higher sulfur deposition. Figure 8 shows the resulting amounts of carbon and sulfur on the catalyst used after each activity test, respectively.

The results from the elemental analysis indicate a reduced tendency for carbon polymerization and coke formation on all the doped NiMo catalysts but in particular for the presulfidized catalyst in a reaction with a co-feed of 1% H_2_S. This agrees with previously discussed resulting catalytic acidity characteristics and activity tests. Decreased acidity, as detected for the doped NiMo catalyst, is generally recognized to suppress the prerequisites of polymerization and hence carbon deposition on the surface. Ce is reported, in several studies for partial oxidation, to decrease the coke formation rate.^15,37^ This is in line with the observed differences in the carbon deposition of the two catalysts. It is the redox pair characteristics of Ce (Ce^IV^/Ce^III^) that are stated to be beneficial in this respect. Ce enhances the adsorption and migration of water and oxygen, leading to an increased gasification of carbon.^31^ However, the resulting effect of this, in relation to the HDO of bio-oils over NiMo/Al_2_O_3_ catalysts, should be further investigated.

## 4 Conclusions

The doping effect of the impregnation of Ce and La prior to thermal pretreatment of δ-Al_2_O_3_ has been investigated with respect to the support characteristics for a NiMo catalyst and its resulting performance and sulfur dependency in the HDO of vanillin. Textural surface characteristics and crystallinity analyzed by N_2_ physisorption and XRD showed that the addition of Ce and La stabilizes the Al_2_O_3_ support. NH_3_ chemisorption revealed less total acidity of the Ce- and La-doped support, and XRD and NH_3_ TPD indicated that this was caused by Ce blockage. The HDO tests indicated enhanced selectivity for direct HDO using the doped NiMo/CeLa/Al_2_O_3_ catalyst, where less CO was formed and increased amounts of cresol were obtained. The addition of sulfur was essential for maintaining the activity of all catalysts tested. Significantly less carbon was formed on the doped NiMo/CeLa/Al_2_O_3_ catalyst, which argues for prolonged activity and lifetime. Overall, the impregnation of Ce and La as a pretreatment step for δ-Al_2_O_3_ to be used as a support for NiMo catalysts demonstrates promising activity characteristics and beneficial performance in the HDO of bio-oils.

## Supporting Information Available

Conversion of vanillin over time in each specified activity test over the different catalysts (PDF)
