# Supplementary material for: Promoting Effect of Ce and La on Ni–Mo/δ-Al2O3 Catalysts in the Hydrodeoxygenation of Vanillin
Source: Energy Fuels. 2024 May 18;38(11):9827–35. doi: 10.1021/acs.energyfuels.4c00898 (PMC11165990; doi:10.1021/acs.energyfuels.4c00898)
Supplement: Supplementary file 1 — ef4c00898_si_001.pdf [file ef4c00898_si_001.pdf]

SUPPORTING INFORMATION

Promoting Effect of Ce and La on Ni-Mo/δ-Al_2O_3 Catalysts in the Hydrodeoxygenation of Vanillin

Tove A. Kristensen[a,b,], Christian P. Hulteberg[a,b], Reine L. Wallenberg[c], Omar Y. Abdelaziz[d,e], Sara Blomberg[a]*

[a]Division of Chemical Engineering, Department of Process and Life Science Engineering, Lund University, SE-221 00 Lund, Sweden
[b]Hulteberg Chemistry & Engineering AB, SE-212 25 Malmö, Sweden
[c]Centre for Analysis and Synthesis/nCHREM, Lund University, SE-221 00 Lund, Sweden
[d]Department of Chemical Engineering, King Fahd University of Petroleum & Minerals, Dhahran 31261, Saudi Arabia
[e]Interdisciplinary Research Center for Refining & Advanced Chemicals, King Fahd University of Petroleum & Minerals, Dhahran 31261, Saudi Arabia

*E-mail: tove.kristensen@ple.lth.se

Number of pages: 1
Number of figures: 1

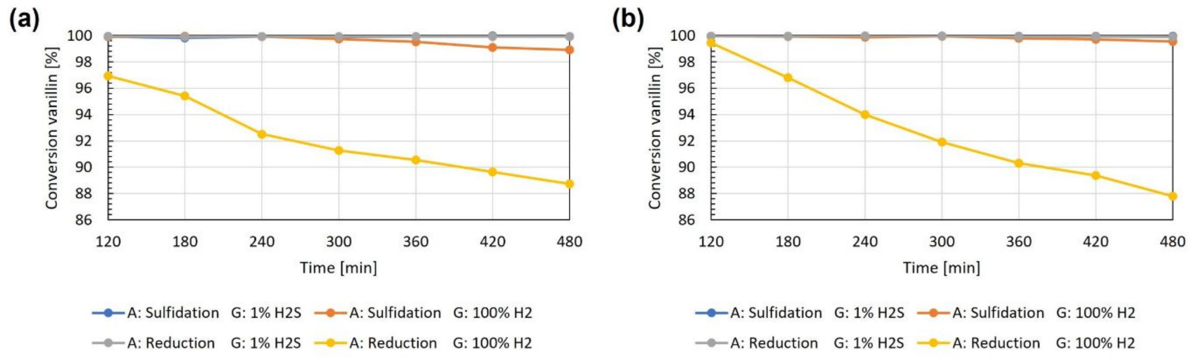

Figure S1. Conversion over time in each specified activity test over (a) NiMo/δ-Al_2O_3 (b) NiMo/CeLa/Al_2O_3. Varying experimental factors used for each test are presented as A (activation method; sulfidation or reduction) and G (gas feed used during HDO; 100% H_2 or 1% H_2S (in 99% H_2)). Experimental conditions: T= 314 °C and P = 5 $bar_{(g)}$.
